# Trends in outcomes of women with myocardial infarction undergoing primary angioplasty—Analysis of randomized trials

**DOI:** 10.3389/fcvm.2022.953567

**Published:** 2023-01-04

**Authors:** Zuzana Motovska, Ota Hlinomaz, Michael Aschermann, Jiri Jarkovsky, Michael Želízko, Petr Kala, Ladislav Groch, Michal Svoboda, Milan Hromadka, Petr Widimsky

**Affiliations:** ^1^Cardiocentre, Third Faculty of Medicine, Charles University and University Hospital Kralovske Vinohrady, Prague, Czechia; ^2^Department of Cardioangiology, International Clinical Research Center, St. Anne’s University Hospital Brno, Brno, Czechia; ^3^Department of Cardiovascular Medicine, First Faculty of Medicine, Charles University, General University Hospital, Prague, Czechia; ^4^Institute of Biostatistics and Analyses Ltd., Faculty of Medicine, Masaryk University, Brno, Czechia; ^5^Department of Cardiology, Institute of Clinical and Experimental Medicine, Prague, Czechia; ^6^Department of Internal and Cardiology, Faculty of Medicine, Masaryk University and University Hospital Brno-Bohunice, Brno, Czechia; ^7^Department of Cardiology, Charles University, University Hospital in Pilsen, Pilsen, Czechia

**Keywords:** myocardial infarction, primary PCI, women, outcome, trends, mortality, therapy management

## Abstract

**Background:**

Sex- and gender-associated differences determine the disease response to treatment.

**Aim:**

The study aimed to explore the hypothesis that progress in the management of STE-myocardial infarction (STEMI) overcomes the worse outcome in women.

**Methods and results:**

We performed an analysis of three randomized trials enrolling patients treated with primary PCI more than 10 years apart. PRAGUE-1,-2 validated the preference of transport for primary PCI over on-site fibrinolysis. PRAGUE-18 enrollment was ongoing at the time of the functional network of 24/7PCI centers, and the intervention was supported by intensive antiplatelets. The proportion of patients with an initial Killip ≥ 3 was substantially higher in the more recent study (0.6 vs. 6.7%, *p* = 0.004). Median time from symptom onset to the door of the PCI center shortened from 3.8 to 3.0 h, *p* < 0.001. The proportion of women having total ischemic time ≤3 h was higher in the PRAGUE-18 (OR [95% C.I.] 2.65 [2.03–3.47]). However, the percentage of patients with time-to-reperfusion >6 h was still significant (22.3 vs. 27.2% in PRAGUE-18). There was an increase in probability for an initial TIMI flow >0 in the later study (1.49 [1.0–2.23]), and also for an optimal procedural result (4.24 [2.12–8.49], *p* < 0.001). The risk of 30-day mortality decreased by 61% (0.39 [0.17–0.91], *p* = 0.029).

**Conclusion:**

The prognosis of women with MI treated with primary PCI improved substantially with 24/7 regional availability of mechanical reperfusion, performance-enhancing technical progress, and intensive adjuvant antithrombotic therapy. A major modifiable hindrance to achieving this benefit in a broad population of women is the timely diagnosis by health professional services.

## Introduction

Women with acute myocardial infarction (MI) have a worse prognosis than men (1–4). This is partly due to the older age of women and age-associated comorbidities. Is also partly explained by other cellular and molecular mechanisms associated with the specific effects of sex-chromosomes complement and of the regulation by gonads hormones (5). In addition to somato-physiological, psycho-social differences play a significant role. Women’s behavior differs from that of men, as evidenced by the persistence of longer times between onset of MI symptoms and the decision to seek medical care in women (6, 7). Sex (biological)- and gender (cultural)- associated differences modify the disease response to treatments (8).

Scientific and societal attention to this issue is reinforced by the growing incidence of MI in younger (ages 35–54) women over the last two decades due to the increased prevalence of risk factors such as diabetes and hypertension, and by the fact that the mortality from heart disease is the leading cause of death in women and is seven times higher than mortality due to breast cancer (9, 10).

Women with ST-segments elevation MI (STEMI) are less likely to receive pharmacological reperfusion and have a worse outcome after fibrinolytic therapy compared to men (11). The approach to the treatment of STEMI has undergone a marked transformation regarding preference for mechanical reperfusion, percutaneous coronary intervention (PCI) network organization that shortens the time to reperfusion, intensification of antithrombotic treatment in the acute phase, and its dynamic adjustment to ischemia and bleeding risks over the long-term. Since women are a special subgroup representing 23 - 40% (12) of STEMI patients, it is important to determine whether directions of treatment strategies translate to offset the unfavorable baseline position of women.

The multicenter randomized PRAGUE (PRimary Angioplasty in patients from General community hospitals transported to catheterization Units compared to Emergency thrombolysis without transport) -1 (13) and -2 (14) studies validated the benefits of timely transport for mechanical reperfusion over on-site fibrinolysis in patients with STEMI and justified regionalization of the 24/7 primary PCI network.

Enrollment for the multicenter randomized PRAGUE-18 (15) study was ongoing when the system of 24/7 PCI centers became fully functional and when primary angioplasty was supported by using intensive antithrombotic medication containing the third-generation P2Y_12_ inhibitor. Changes in the PRAGUE-18 study design and therapy mirrored progress in STEMI management during the almost two decades that elapsed between studies. Exclusion criteria for the PRAGUE-1, -2, and -18 studies reflected contraindications of evaluated treatments. The studies provide all-comers data that is not biased by selection and comes in the form of controlled randomized clinical investigation(s).

Presented work explores the hypothesis that improvement in PCI network organization, progress in procedural and instrumental aspects of mechanical reperfusion (16), and adjuvant antithrombotic therapy for STEMI have offset worse outcomes in women.

## Materials and methods

The presented work analyses data of female patients enrolled in three randomized studies over almost two decades involving patients undergoing primary PCI. The PRAGUE-1 study enrolled patients from June 1997 until March 1999, the PRAGUE-2 study conducted randomization between September 1999 and January 2002, and the PRAGUE-18 study randomized patients from April 2013 to May 2016. All study designs were published previously (Supplementary Figure 1 and Supplementary Table 1) (13–15). In the PRAGUE-1 and -2 studies, STEMI patients admitted to hospital (emergency departments) without an on-site cardiac catheterization laboratory were randomized to (1) on-site thrombolysis or (2) transported to hospitals with primary PCI capability. Presented analyses focused on female primary PCI patients only. Almost two decades after the PRAGUE-1 and -2 studies, the PCI network had changed substantially, and STEMI patients in the PRAGUE-18 study were transported directly to the catheterization laboratories for primary PCI and randomized to ticagrelor or prasugrel as the standard of care.

Experienced interventional cardiologists (at all study sites, which were high-volume PCI centers) performed the coronary intervention procedures (primary PCI), and made procedural decisions, including (contemporary) stent selection. In the PRAGUE-1, -2 study, per protocol, femoral access was used for PCI, which was the standard at that time and involved antithrombotic therapy consisting of aspirin, clopidogrel, and heparin. Clopidogrel was later replaced by ticagrelor or prasugrel in the PRAGUE-18 study. In all studies, GP IIb/IIIa inhibitors were available at the discretion of treating interventional cardiologists, during the primary PCI procedure, as a bailout therapy. After hospital discharge, low-risk patients in the PRAGUE-18 study were allowed to switch, after approval of the treating physician, from prasugrel or ticagrelor to clopidogrel for economic reasons (17). Mortality at 30 days after enrollment was the primary endpoint in the PRAGUE-2 study and an assessed endpoint in the PRAGUE-1 and PRAGUE-18 studies. Definitions of study endpoints are detailed in Supplementary Table 2.

The MI diagnosis fundamentally changed over the period used for this analysis, mainly due to the mandatory use of highly sensitive (immunoassays for detection of) troponins. In the PRAGUE-1 and -2 studies, MI was defined using the WHO/MONICA definition, which was based on the presence of symptoms and electrocardiographic patterns and/or elevation of cardiac markers (enzyme creatine kinase) in the blood. While in the PRAGUE-18 study, the Third universal definition of MI was used. Substantial differences in sensitivity and specificity to diagnose the same clinical entity according to these definitions, did not allow a comparison of the incidence of new spontaneous reinfarction over the evaluated period (18).

All three studies were approved by multicenter ethics committees and the relevant institutional ethics committees of participating sites and were carried out in agreement with the Declaration of Helsinki for Good Clinical Practice. The PRAGUE-1, -2, and -18 studies were purely academic projects without any commercial support and were designed and coordinated by Cardiocenter of the Third Faculty of Medicine, Charles University, and the Kralovske Vinohrady University Hospital in Prague (Czechia).

Patient and public involvement. All female patients included in the presented analysis signed informed consent with participation in specified randomized studies.

### Statistical analysis

Categorical variables were described by absolute and relative frequencies. Relative frequencies were calculated using valid data. Continuous parameters were described using verified *N* values, means (standard deviation, SD), and medians (5th and 95th percentiles). Differences between categorical variables were tested using the Pearson Chi-Square test or Fisher exact test. Differences between continuous variables were tested using the Mann–Whitney *U* test. Binary logistic regression [with odds ratios (OR) and 95% confidence intervals (C.I.)] was used for outcome analyses (i.e., procedural and clinical). Prediction of 30-day death was also adjusted by Killip class, age and history of previous MI using multivariate logistic regression model. Level of statistical significance was set up as α = 0.05. The analysis was performed in IBM SPSS Statistics 24.0.0.1.

## Results

### Study population

Baseline and procedural characteristics are detailed in Table 1. The proportion of women in the primary PCI arms increased from 14.5% (N 29/200) in PRAGUE-1 and 15.3% (N 130/850) in PRAGUE-2 to 24.3% (N 299/1230) in the PRAGUE-18 study. Except for the age of 18 years as the youngest, there were no age restrictions for older patient enrollment. Women in the PRAGUE-1, -2 studies were older (Median [5th; 95th percentile] 69.0 [49.0; 80.0] years) than those in the PRAGUE-18 study (64.9 [46.1; 83.1], *p* = 0.021). The prevalence of diabetes and hypertension comorbidities were comparable. In the PRAGUE-18 study, more women were active cigarette smokers. The proportion of female patients with initially severe acute heart failure (Killip ≥ 3) was significantly higher in the PRAGUE-18 study (0.6 vs. 6.7%; OR [95% C.I.] 10.97 [1.46–82.52] *p* = 0.004).

**TABLE 1:** Baseline and procedural characteristics.

		Women cohorts		*P*-value
		PCI-PRAGUE-1, and -2 (*N* = 159)	PRAGUE-18 (*N* = 299)	
Age (years)	Mean (SD) Median (5th–95th perc.)	66.7 (10.7); 69.0 (49.0; 80.0)	64.4 (10.7); 64.9 (46.1; 83.1)	0.006
Smoking status	Current smoker	21 (22.1%)	151 (50.5%)	<0.001
	Ex-smoker	9 (9.5%)	25 (8.4%)	
Arterial hypertension		81 (63.3%)	189 (63.2%)	0.989
Diabetes mellitus		40 (31.5%)	83 (27.8%)	0.436
Previous MI		22 (13.9%)	19 (6.4%)	0.007
Killip class	1	122 (79.2%)	257 (86.0%)	<0.001
	2	31 (20.1%)	22 (7.4%)	
	3	1 (0.6%)	7 (2.3%)	
	4	0 (0.0%)	13 (4.3%)	
	1–2	153 (99.4%)	279 (93.3%)	0.004
	3–4	1 (0.6%)	20 (6.7%)	
LV EF (%)^4^	Mean (SD) Median (5th–95th perc.)	49.0 (10.5); 50.0 (33.0; 65.0)	48.1 (10.6); 50.0 (30.0; 65.0)	0.357
IRA	LAD	68 (42.8%)	119 (39.8%)	0.538
	Cx	24 (15.1%)	50 (16.7%)	0.652
	RCA	57 (35.8%)	130 (43.5%)	0.114
	LM	1 (0.6%)	2 (0.7%)	0.999
No. of diseased vessels	0	7 (4.5%)	0 (0.0%)	<0.001
	1	52 (33.5%)	134 (45.0%)	
	2	38 (24.5%)	101 (33.9%)	
	3	55 (35.5%)	63 (21.1%)	
	>1	93 (60%)	164 (55%)	0.311
Left main disease		3 (1.9%)	8 (2.7%)	0.755
Femoral access		159 (100%)	108 (36.1%)	<0.001
TIMI flow before PCI	0	88 (59.9%)	148 (50.0%)	0.272
	1	12 (8.2%)	32 (10.8%)	
	2	24 (16.3%)	61 (20.6%)	
	3	23 (15.6%)	55 (18.6%)	
	0	88 (59.9%)	148 (50.0%)	0.050
	1–3	59 (40.1%)	148 (50.0%)	
TIMI flow after PCI	0	7 (5.1%)	1 (0.3%)	<0.001
	1	6 (4.4%)	2 (0.7%)	
	2	7 (5.1%)	10 (3.4%)	
	3	116 (85.3%)	283 (95.6%)	

SD, standard deviation; Cx, circumflex coronary artery; IRA, infarct-related artery; LAD, left anterior descending coronary artery; LM, left main; LV EF, echocardiographic left ventricle ejection fraction; MI, myocardial infarction; PCI, percutaneous coronary intervention; RCA, right coronary artery; TIMI, Thrombolysis in Myocardial Infarction. TD, time delay; “Symptoms to door” means: From symptom onset to the door of PCI center. Categorical parameters are described by absolute (relative) frequencies. Differences are tested by Pearson Chi Square test or Fisher exact test. Continuous parameters are described by mean (SD), median (5. and 95. percentiles). Differences are tested by Mann–Whitney test. Descriptive statistics are calculated from valid data.

^1^Analysis was based on available data in PCI-PRAGUE 2 (*N* = 127) and PRAGUE 18 (*N* = 253).

^2^Women with non-STEMI treated with primary angioplasty and randomized into the PRAGUE 18 study were excluded from this analysis.

^3^Analysis was based on available data in PCI-PRAGUE 1,2 (*N* = 125) and PRAGUE 18 (*N* = 254).

^4^Analysis was based on available data in PRAGUE 2 (*N* = 101) and PRAGUE 18 (*N* = 217).

### Delay of reperfusion

The median time from onset of symptoms to the door of a PCI center shortened significantly from 3.8 h in the PRAGUE-2 study to 3.0 h in the PRAGUE-18 study, *p* < 0.001. The mean (SD) duration of in-hospital delay (from the door) to reperfusion was longer in the PRAGUE-18 study [0.7 (1.7) hours vs. 0.5 (0.2) hours in women undergoing primary PCI in the first two PRAGUE studies, *p* < 0.001] (Table 2).

**TABLE 2:** Time delay to reperfusion.

	Women cohorts			*P*-value^1^	*P*-value^2^
	(PCI) PRAGUE-1, 2 (*N* = 159)	(PCI) PRAGUE-2 (*N* = 130)	PRAGUE-18* (*N* = 277)		
Symptoms—door (hours)	4.3 (2.0); 3.8 (1.9; 8.0)	4.4 (2.1); 3.8 (1.9; 8.0)	4.1 (3.6); 3.0 (1.0; 10.3)	<0.001	<0.001
Door—balloon (hours)	0.5 (0.2); 0.4 (0.3; 0.8)	0.4 (0.2); 0.4 (0.3; 0.8)	0.7 (1.7); 0.3 (0.1; 2.0)	<0.001	0.002
Symptoms—balloon (hours)	4.7 (1.9); 4.2 (2.5; 7.9)	4.8 (2.0); 4.3 (2.5; 7.9)	4.7 (3.7); 3.5 (1.3; 11.0)	0.003	0.003
≤1.0 h	0 (0.0%)	0 (0.0%)	5 (2.0%)	<0.001	<0.001
1.1–3.0 h	18 (14.4%)	15 (14.6%)	101 (40.4%)		
3.1–6.0 h	80 (64.0%)	65 (63.1%)	76 (30.4%)		
>6.0 h	27 (21.6%)	23 (22.3%)	68 (27.2%)		
≤3.0 h	18 (14.4%)	15 (14.6%)	106 (42.4%)	<0.001	<0.001
>3.0 h	107 (85.6%)	88 (85.4%)	144 (57.6%)		

PCI, percutaneous coronary intervention; Categorical parameters are described by absolute (relative) frequencies. Differences are tested by Pearson Chi Square test or Fisher exact test. Continuous parameters are described by mean (SD), median (5. and 95. percentiles). Differences are tested by Mann–Whitney test. Descriptive statistics are calculated from valid data.

*Women with NSTEMI are excluded from this analysis.

^1^Comparison of (PCI) PRAGUE-1, 2 and PRAGUE-18.

^2^Comparison of (PCI) PRAGUE-2 and PRAGUE-18.

**FIGURE 1:**
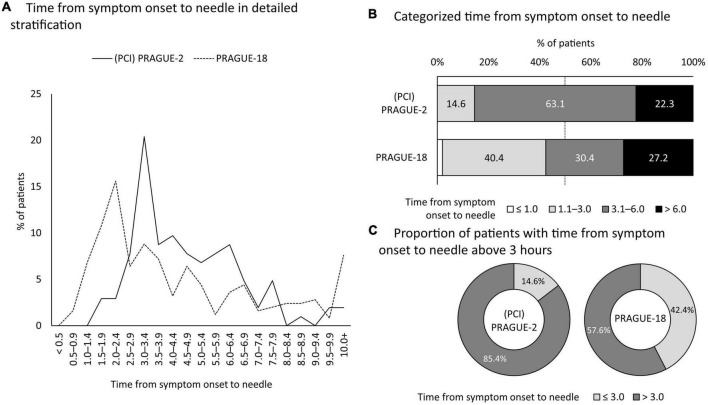
Trends in time delay to reperfusion by primary angioplasty. Based on the PRAGUE-2 and PRAGUE-18 studies. PCI, percutaneous coronary intervention.

As depictured on the Figure 1, the proportion of female patients having total ischemic times [from the onset of symptoms to reperfusion (balloon)] ≤3 h was almost three times higher in the PRAGUE-18 study (42.4 vs. 14.6%, *p* < 0.001; OR [95% C.I.] 2.65 [2.03–3.47]). However, the percentage of women with time-to-reperfusion >6 h was still significant (22.3% in PRAGUE-2 vs. 27.2% in PRAGUE-18) (Figure 1B; Table 2).

### Angiography and angioplasty (primary PCI)

In contrast to the first studies, where femoral access was used in all patients (see Section “Materials and methods”), in the PRAGUE 18 study, this was only in 36.1% of women (Table 1). The representation of infarct-related arteries was comparable between the studies, as was the incidence of the left main disease. Multivessel disease was present in 60% of women in the PRAGUE-1 and -2 studies and in 55% in the PRAGUE 18 study (*p* = 0.311). The probability for an initial non–TIMI (Thrombolysis in Myocardial Infarction) flow 0 (OR [95% C.I.] 1.49 [1.0–2.23]), and for an optimal procedural result of primary PCI increased significantly in the newer study (4.24 [2.12–8.49], *p* < 0.001) (Table 1).

### Prognosis

**FIGURE 2:**
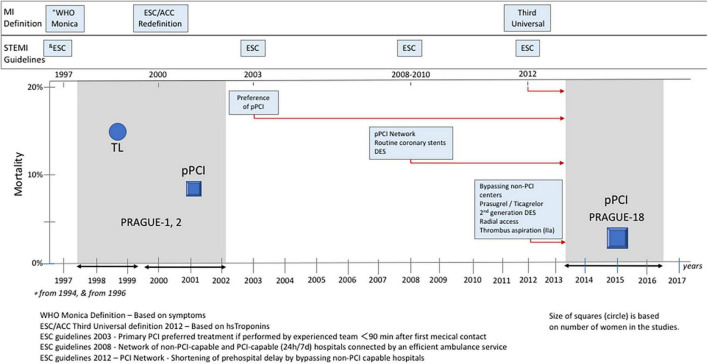
Trends in 30-day mortality.

**FIGURE 3:**
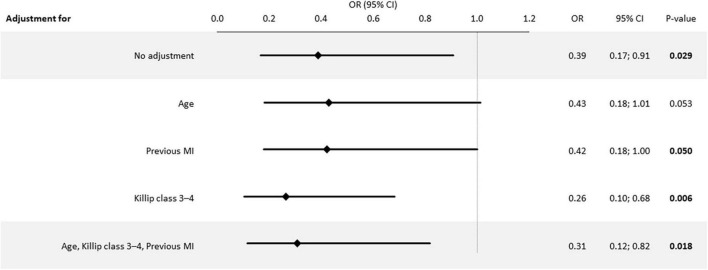
Adjusted Risk of 30-day mortality in the PRAGUE-18 vs. PCI-PRAGUE-1, -2 studies. OR, Odds ratio; CI, confidence interval; MI, myocardial infarction; age was entered as continuous variable in the model.

The mortality trend in women with STEMI treated with primary PCI is shown in Figure 2. The risk of death within 30-days after the index event changed substantially from 8.2% in women undergoing primary PCI in the PRAGUE-1 and -2 studies to 3.3% in women enrolled to the PRAGUE-18 study. The relative risk of 30-day mortality of women randomized to primary PCI decreased by 61% (0.39 [0.17–0.91], *p* = 0.029). The decrease in mortality is even more apparent after adjustment for basic characteristics (age, Killip class, previous MI) different between datasets (0.31 [0.12–0.82], *p* = 0.018) (Figure 3).

The risk of stroke during the first month after STEMI was and remained low. There was one woman (0.6%) in the PRAGUE-1 and -2 studies who experienced a stroke, while there were no strokes in women in the PRAGUE-18 study within the 30-days (Table 3).

**TABLE 3:** Outcomes—procedural and clinical at 30 days.

		Women cohorts		*P*-value	OR (95% CI)	*P*-value
		PCI-PRAGUE 1, 2 (*N* = 159)	PRAGUE 18 (*N* = 299)		Ref. cat: PCI-PRAGUE 1,2	
PCI result^#^	Optimal	114 (82.6%)	282 (95.3%)	<0.001	4.24 (2.12; 8.49)	<0.001
	Sub-optimal	11 (8.0%)	10 (3.4%)		0.40 (0.17; 0.97)	0.044
	Unsuccessful	13 (9.4%)	4 (1.4%)		0.13 (0.04; 0.41)	<0.001
Death		13 (8.2%)	10 (3.3%)	0.024	0.39 (0.17; 0.91)	0.029
Stroke		1 (0.6%)	0 (0.0%)	0.347	−	−

PCI, percutaneous coronary intervention; Categorical parameters are described by absolute (relative) frequencies. Relative frequencies are calculated from valid data. Differences are tested by Pearson Chi Square test or Fisher exact test and described using OR (odds ratio) and its 95% CI (Confidence interval). ^#^The result of the PCI procedure was assessed as optimal, suboptimal, or unsuccessful by the attending interventional cardiologist.

## Discussion

Sex and gender affect the body comprehensively. In the context of cardiovascular diseases, there are significant differences between men and women relative to the heart, brain, vascular system, liver, kidneys, and drug metabolism and excretion (5, 19). These differences are reflected in the manifestation, pathophysiology, response to therapy, and therapy complications of myocardial infarction (20).

Large dataset analyses have consistently and persistently demonstrated less favorable outcomes in women with acute MI as compared to men, particularly those with STEMI, and especially those under the age of 60 years (1–4, 21–24).

The present work analyzed three randomized trials, i.e., the PRAGUE-1, -2, and -18 studies, reflecting changes in the treatment of MI caused by complete coronary artery occlusion over a 19-year period, i.e., 1997 (the start of randomization to the PRAGUE-1 study) to 2016 (the end of PRAGUE-18 enrollment) (Figure 2). The PRAGUE-1 and -2 studies documented the survival benefit of transport for mechanical reperfusion over on-site thrombolysis (13, 14, 25). These priority evidence triggered the regionalization of primary angioplasty *via* a network of 24/7 PCI centers (26). Registry data documented that adherence to the preference for primary angioplasty over thrombolysis significantly reduced the number of STEMI patients without reperfusion therapy (27). In addition, the implemented changes in the organization of care led to bypassing non-PCI hospitals by emergency medical services. The goal was to make the most effective reperfusion therapy available at the earliest point in time. This achievement was confirmed by a significant shortening of the total ischemic time in women enrolled in the PRAGUE-18 study (Table 2; Figure 1). Where, in addition, primary angioplasty was supported by the most intensive antiplatelet therapy with prasugrel or ticagrelor. Direct transport of women to the PCI center led to extending the mean in-hospital delay (from the door to the balloon) in the PRAGUE-18 study. This was conditioned by the significantly higher proportion of unstable female patients in this study. Furthermore, even in the case of direct transport to a hospital with 24/7 PCI capability, some patients are, because of diagnostic uncertainty, which is a frequent problem of healthcare providers in women, first evaluated at the emergency department (28). Nevertheless, the median total duration of ischemia was substantially shorter in the recent study.

Over the time between the PRAGUE-1 and PRAGUE-18 studies, considerable progress was made in coronary intervention. Radial access began and increased to the point that it was used in almost two-thirds of the women in the PRAGUE-18 study. In the setting of primary angioplasty associated with the highest risk of access site complication, use of radial access represents a crucial complication preventive measure. Especially in women in the era of intense adjuvant antiplatelets, while women are at high risk of major bleeding from the femoral access site and radial access lowered this risk two- to three-fold (29, 30).

Stent technology has evolved from bare-metal stents through a series of new-generation drug-eluting stents in which we saw improvements in both drug elution and stent design (31). New-generation drug-eluting stents were preferred for primary PCI at the time of the PRAGUE-18 study. Women tend to have smaller sizes and significantly more tortuous coronary arteries, and more vulnerability to dissection, which can represent a technical challenge in the setting of coronary interventions (32). Fundamental developments in the percutaneous coronary intervention armamentarium led to increased procedural safety, effectiveness, and success in women (16, 31).

Significant reductions in total ischemic time, procedural advancement in primary PCI supported by potent antiplatelet therapy resulted in optimal reperfusion in the PRAGUE-18 being several times higher than in the PRAGUE-1, and -2 studies (OR [95% C.I.] 4.24 [2.12; 8.49], *p* < 0.001). While, primary angioplasty postprocedural results independently determine the prognosis in women with STEMI (33).

A key feature of the revolutionary changes in STEMI treatment during the times between studies was a remarkable decline in the risk of death, with a decrease in the relative risk of 61%. This was despite there being substantially more women with initially more advanced acute heart failure (Killip ≥ 3) in the PRAGUE-18 study compared to the PRAGUE-1,-2 studies (i.e., 6.7 vs. 0.6%).

We have detailed data on bleeding complications from the PRAGUE-18 study. Unfortunately, not from the PRAGUE 1 and 2 studies. However, presented results confirm that there is no *a priori* reason to fear bleeding in women treated with primary PCI for STEMI when using highly potent third-generation P2Y_12_ inhibitors. The increased incidence of bleeding, which accompanies intense inhibition of platelet reactivity, does not reduce the survival benefits in women with the highest thrombotic risk. The PRAGUE-18 sub-study showed comparable platelet reactivity before and after prasugrel or ticagrelor therapy in women and men matched on propensity score (Supplementary Figure 2). Gender should not influence the choice of P2Y_12_ inhibitors for STEMI; however, meticulous weight- and age-adjusted doses of GPIIb/IIIa inhibitors, heparins, and prasugrel are required (20).

Advances in STEMI treatment have been shown to overcome the unfavorable prognostic effect of being female, *per se*, as a complex and specific biological variable, in those that arrive early. Women who arrive at PCI-centers within 120 min of symptom onset have the comparable outcome as identically risk men (33); however, ischemia, in women, lasting longer than 3 h makes ischemia-associated consequences more severe (34). These findings underlay the critical relationship between primary PCI benefits and timely reperfusion in women with totally occluded coronary artery.

Despite improvement, 57.6% of women in the PRAGUE-18 study still had symptom-to-reperfusion time >3 h. The proportion of women with reperfusion more than 6 h after the onset of symptoms remain unacceptably high even after two decades of advancement, as this time delay was present in every fourth woman enrolled in the PRAGUE studies (Figure 1B). The female gender, which manifests as a specific set of psycho-social and cultural norms, still represents a barrier to seeking timely medical care after the onset of MI symptoms. Symptom to reperfusion delay in women has not been shortened in advanced economies or using specialized education objectives (i.e., public, by health professional). Nor has it been shortened relative to the severity of symptoms (35). As such, female gender is, in this context, an independent variable for the persistence of patient delay to reperfusion in STEMI and has remained unmodifiable for decades.

Our analysis confirmed that the representation of women in randomized STEMI trials is low, especially at younger ages (36). The age-related underestimation of symptoms and the diagnostic preference for alternative causes of ST-segment elevation during the acute phase (which is often prehospital) need to be recognized (37). Female patients with STEMI have longer prehospital healthcare delays, hospital delays, and total healthcare delays compared with male patients with STEMI (28). Significant modifiable factors for timely reperfusion in women are (1) an immediate diagnosis at the first contact with medical service professionals, (2) an underestimation of symptoms in younger women, and (3) not knowing the range of symptom diversity in women (38, 39). New initiatives are aimed at the necessity of reducing medical care-related delays, as well as raising awareness among healthcare providers regarding sex- and gender-related disparities in STEMI care and increased awareness of evidence-based therapy in women (40).

Research efforts to further improve the prognosis of women with MI caused by a total thrombotic occlusion of a coronary artery focus on reducing the risk of developing microvascular obstructions and intensifying cardioprotective strategies. Women are known to be at high risk of developing heart failure after STEMI; however, so far, promising cardioprotective strategies have not yet been shown to affect prognoses (41). Nevertheless, a comprehensive therapeutic approach, focusing on both infarct size and microvascular obstruction, could help translate cardioprotective strategies into improved clinical outcomes in female STEMI patients (34).

## Limitations

The analysis compares treatments in the populations of three randomized trials. Considering the compared populations, the expected limitation involves the heterogeneity of study groups. Heterogeneity relative to basic characteristics was only present in the age, history of MI, and Killip class at admission of the compared patients. However, adjustments to the baseline variables showed that the age difference did not affect the 30-day risk of mortality (age in years OR [95% C.I.] 1.04 [0.99–1.09], *p* = 0.147).

The women in the PRAGUE-1 and 2 studies and those in the PRAGUE-18 study did not differ in the proportion of risk factors (i.e., diabetes and hypertension) nor in the representation of infarction-related arteries, presence of left main and multivessel disease; however, in contrast, women in the PRAGUE-18 study, who had a significantly lower risk of 30-day mortality, also had a several times higher incidence of advanced acute heart failure at admission.

Nonetheless, our analysis is based on the limited number of women enrolled in the three studies, which accurately reflects the generally lower representation of women in STEMI trials (see Section “Discussion”). On a positive note, the data used in the studies were very controlled, and the study population, treatment algorithm, and endpoints were precisely defined. Data from randomized controlled studies represent the best evidence to guide therapies. Analyzes of even large populations, however, non-randomized, retrospective, and observational studies are potentially affected by important bias and might not represent a real effect (42). Furthermore, it should be noted that the significant benefit related the modern therapeutic management of STEMI patients was documented by a hard endpoint, i.e., reduction of mortality.

## Conclusion

Evidence-based progress in the management of STEMI, changes in the extent of the primary PCI network, procedural instrumentation and technique, and adjuvant antiplatelet therapy have provided improvements in primary angioplasty results and resulted in substantial improved prognoses for women with acute myocardial infarction. A major modifiable hindrance to achieving this benefit in a broad population of women is the timely diagnosis by health professional services.

## What is already known about this subject?

Sex–and gender–associated differences modify the disease response to treatments. Data from randomized controlled studies represent the best evidence to guide therapies. Analysis of the effect of treatment for each gender separately can improve the understanding of the importance of disease specific aspects in men and women.

## What does this study add?

Evidence-based progress in the management of STEMI have been shown to overcome the unfavorable prognostic effect of being female, *per se*, as a complex and specific biological variable. The significant benefit related the modern therapeutic management of STEMI female patients was documented by a hard endpoint, i.e., reduction of mortality. However, the proportion of women with time to reperfusion more than 6 h after the onset of symptoms remain unacceptably high even after two decades of advancement.

## How might this impact on clinical practice?

A major modifiable hindrance to achieving the benefit in a broad population of women is the timely diagnosis by health professional services. New initiatives are aimed at the necessity of reducing medical care-related delays, as well as raising awareness among healthcare providers regarding sex- and gender-related disparities in STEMI care and increased awareness of evidence-based therapy in women.

## Data availability statement

The data analyzed in this study is subject to the following licenses/restrictions. Requests to access these datasets should be directed to JJ, jarkovsky@iba.muni.cz.

## Ethics statement

The studies involving human participants were reviewed and approved by the ethics committees approved three multicentre studies of all participating centers. The patients/participants provided their written informed consent to participate in this study.

## Author contributions

ZM: study conception and design and responsible for the overall content as guarantor. PW: revising the manuscript critically for important intellectual content. PW and ZM: design and analysis of the studies. PW, ZM, OH, MA, MŽ, RM, LG, and MH: data collection. ZM, JJ, and MS: analysis and interpretation of results. ZM and JJ: draft manuscript preparation. All authors reviewed the results and approved the final version of the manuscript.
