# Supplementary material for: Trends in outcomes of women with myocardial infarction undergoing primary angioplasty—Analysis of randomized trials
Source: Front Cardiovasc Med. 2023 Jan 4;9:953567. doi: 10.3389/fcvm.2022.953567 (PMC9845716; doi:10.3389/fcvm.2022.953567)
Supplement: Supplementary file 1 [file Data_Sheet_1.PDF]

TRENDS IN OUTCOME OF WOMEN WITH MYOCARDIAL INFARCTION UNDERGOING PRIMARY ANGIOPLASTY.

– ANALYSIS OF RANDOMIZED TRIALS

Motovska Z. et al.

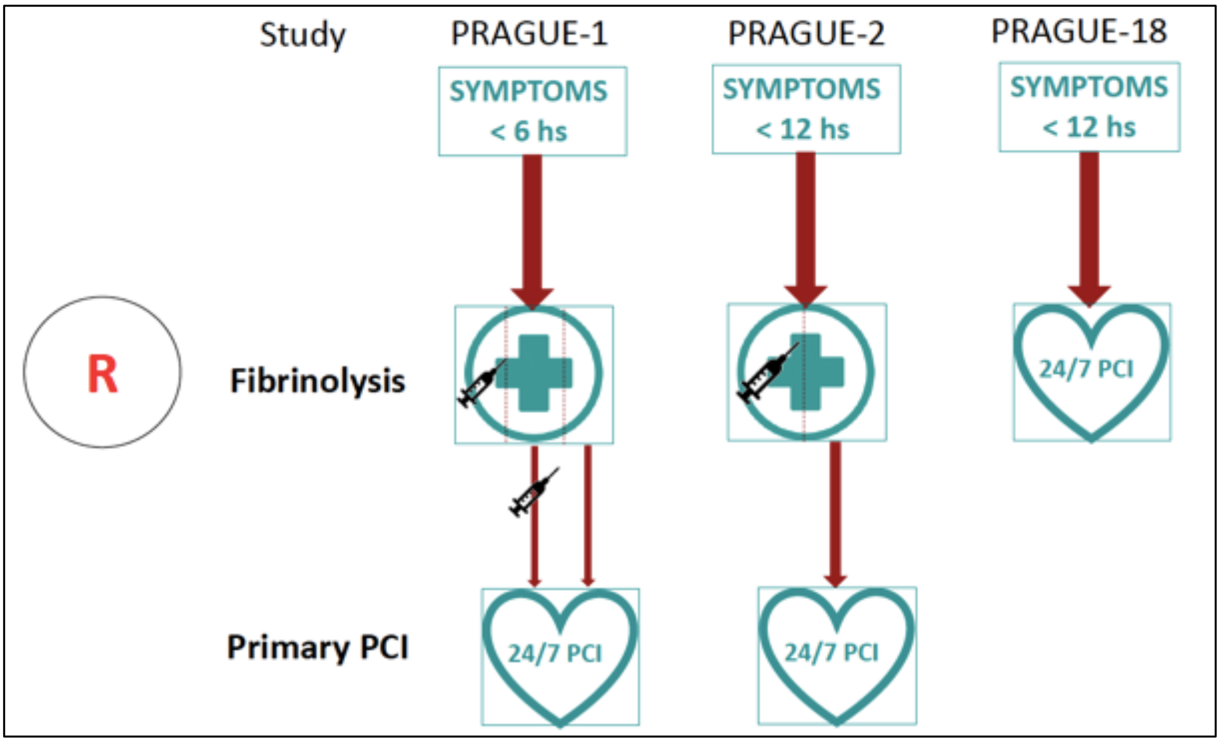

Figure S1. Study Designs

PRAGUE-1 study	PRAGUE-2 study	PRAGUE-18 study
**Inclusion criteria**		
- AMI (ST elevations >1 mm in at least two leads or a new bundle branch block on initial ECG),<br>- Less than 6 h after onset of symptoms,<br>- Time to angioplasty centre <60 min,<br>- Feasibility to begin the transport to the centre within 30 min of randomization,<br>- Signed written informed consent.	- AMI (ST elevations > 1 mm in at least two leads or a new bundle branch block on initial ECG),<br>- Within <12 h from the onset of symptoms,<br>- Distance to PCI centre <120 km,<br>- Feasibility to begin transport within 30 min after randomization,<br>- Signed written informed consent.	- AMI (ST elevation on two related leads at a minimum, by more than 1 mm, or ST depression on three leads at a minimum, by more than 2 mm, or a new bundle branch block),<br>- Emergent (within 120 min of admission to the 24/7PCI center) CAG / primary PCI,<br>- Signed informed consent.
**Exclusion criteria**		
- Contraindications to thrombolysis,<br>- Absence of bilateral femoral artery pulsations.	- Contraindication to thrombolysis (ischaemic stroke within previous 12 months, haemorrhagic stroke at any time, intracranial tumour, active internal bleeding, aortic dissection),<br>- Absence of bilateral femoral artery pulsations.	- History of stroke,<br>- Serious bleeding within the past 6 months,<br>- Indication for long-term oral anticoagulation therapy,<br>- Administration of clopidogrel ≥300 mg or any other antiplatelet medication (except aspirin and a lower dose of clopidogrel) before randomization,<br>- Aged >75 years with a body weight <60 kg (ie, the presence of both parameters),<br>- Moderate or severe hepatic function disorder,<br>- Concomitant treatment with a strong CYP3A4 inhibitor,<br>- Known hypersensitivity to prasugrel or ticagrelor.

Table S1. The PRAGUE studies Inclusion/Exclusion criteria

PRAGUE-1 study	PRAGUE-2 study	PRAGUE-18 study
**Definitions**		
Death: death from any cause within 30 days of randomization.	Death was defined as death from any cause within 30 days of randomization.	Death was defined as a summary of death from any cause.
Re-infarction: Recurrent myocardial infarction was defined as recurrent symptoms of myocardial infarction with a more than double increase in the level of creatine kinase MB and / or new electrocardiographic changes.	Reinfarction: Recurrent symptoms of ischemia with new electrocardiographic changes and a rise in CK-MB.	Re-infarction was defined according to the Third Universal Definition of Myocardial Infarction.
Stroke was defined as any new neurological deficit lasting >24 h.	Stroke was defined as any new neurologic deficit lasting >24 h.	Stroke was defined as a rapid onset of a new neurological deficit caused by an ischemic or hemorrhagic central nervous system event with symptoms lasting at least 24 hours from their onset or leading to death.
Procedural success was defined as TIMI-3 flow and < 50% stenosis after intervention	Optimal procedural success was defined as TIMI-3 flow and <30% stenosis after the intervention.<br><br>Partial success (suboptimal result) was defined as TIMI-2 flow and/or >30% residual stenosis.	Optimal postprocedural result: TIMI grade 3 flow in the infarct-related artery.
**Endpoints**		
Primary end point: Mortality, reinfarction and stroke at 30 days.	Primary end point: Death from any cause within 30 days after randomization.<br><br>Secondary end point: Death/non-fatal reinfarction/non-fatal stroke at 30 days.	Primary end point: All-cause death, reinfarction, stroke, serious bleeding requiring transfusion or prolonging hospitalization, or urgent target vessel revascularization within 7 days after randomization or at discharge if before the seventh day.<br><br>Key secondary efficacy end point: Cardiovascular death, nonfatal myocardial infarction, or stroke during the follow-up period.

Table S2. The PRAGUE studies Definitions

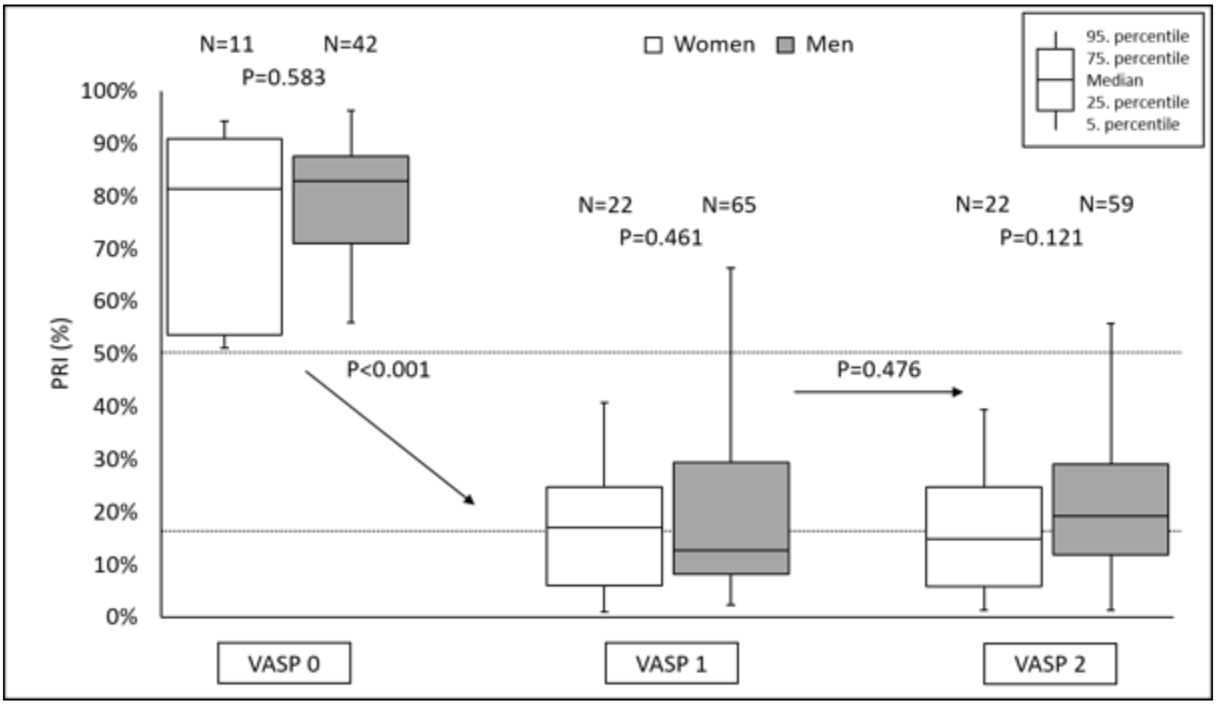

Figure S2. Comparison of Platelet reactivity before and after antiplatelet therapy in women and men with acute myocardial infarction treated with primary percutaneous coronary intervention.

Compared groups are matched (factors: age, diabetes, active cigarette smoking) by propensity score.

VASP 0 – baseline (before antiplatelet agent) platelet reactivity assessed by flow cytometry vasodilator-stimulated phosphoprotein (VASP) phosphorylation state, and expressed as Platelet reactivity index (PRI, %).

VASP 1 – 24±4 hours after loading dose of Prasugrel 60 mg or Ticagrelor 180 mg.

VASP 2 – at discharge on maintenance dose of Prasugrel 10 mg daily or Ticagrelor 90 mg twice a day.

References:

1. Widimsky P, Groch L, Zelizko M, et al. Multicentre randomized trial comparing transport to primary angioplasty vs immediate thrombolysis vs combined strategy for patients with acute myocardial infarction presenting to a community hospital without a catheterization laboratory. The PRAGUE study. *Eur Heart J* 2000;21: 823–31.
2. Widimský P, Budesínský T, Vorác D, Groch L, Zelízko M, Aschermann M, Branny M, St'ásek J, Formánek P; 'PRAGUE' Study Group Investigators. Long distance transport for primary angioplasty vs immediate thrombolysis in acute myocardial infarction. Final results of the randomized national multicentre trial--PRAGUE-2.*Eur Heart J*. 2003;24(1):94-104. doi: 10.1016/s0195-668x(02)00468-2.
3. Motovska Z, Hlinomaz O, Miklik R, Hromadka M, Varvarovsky I, Dusek J, Knot J, Jarkovsky J, Kala P, Rokyta R, Tousek F, Kramarikova P, Majtan B, Simek S, Branny M, Mrozek J, Cervinka P, Ostransky J, Widimsky P; PRAGUE-18 Study Group. Prasugrel Versus Ticagrelor in Patients With Acute Myocardial Infarction Treated With Primary Percutaneous Coronary Intervention: Multicenter Randomized PRAGUE-18 Study. *Circulation*.;134(21):1603-1612. doi: 10.1161/CIRCULATIONAHA.116.024823.
4. Motovska Z, Hlinomaz O, Kala P, Hromadka M, Knot J, Varvarovsky I, Dusek J, Jarkovsky J, Miklik R, Rokyta R, Tousek F, Kramarikova P, Svoboda M, Majtan B, Simek S, Branny M, Mrozek J, Cervinka P, Ostransky J, Widimsky P; PRAGUE-18 Study Group. 1-Year Outcomes of Patients Undergoing Primary Angioplasty for Myocardial Infarction Treated With Prasugrel Versus Ticagrelor. *J Am Coll Cardiol*. 2018;71(4):371-381. doi: 10.1016/j.jacc.2017.11.008.
5. Thygesen K, Alpert JS, Jaffe AS, Simoons ML, Chaitman BR, White HD; Writing Group on the Joint ESC/ACCF/AHA/WHF Task Force for the Universal Definition of Myocardial Infarction, Thygesen K, Alpert JS, White HD, Jaffe AS, Katus HA, Apple FS, Lindahl B, Morrow DA, Chaitman BA, Clemmensen PM, Johanson P, Hod H, Underwood R, Bax JJ, Bonow RO, Pinto F, Gibbons RJ, Fox KA, Atar D, Newby LK, Galvani M, Hamm CW, Uretsky BF, Steg PG, Wijns W, Bassand JP, Menasché P, Ravkilde J, Ohman EM, Antman EM, Wallentin LC, Armstrong PW, Simoons ML, Januzzi JL, Nieminen MS, Gheorghiade M, Filippatos G, Luepker RV, Fortmann SP, Rosamond WD, Levy D, Wood D, Smith SC, Hu D, Lopez-Sendon JL, Robertson RM, Weaver D, Tendera M, Bove AA, Parkhomenko AN, Vasilieva EJ, Mendis S; ESC Committee for Practice Guidelines (CPG). Third universal definition of myocardial infarction. Eur Heart J. 2012;33:2551-67. doi: 10.1093/eurheartj/ehs184.
